# Key Components of Antenatal Lifestyle Interventions to Optimize Gestational Weight Gain: Secondary Analysis of a Systematic Review

**DOI:** 10.1001/jamanetworkopen.2023.18031

**Published:** 2023-06-16

**Authors:** Cheryce L. Harrison, Mahnaz Bahri Khomami, Joanne Enticott, Shakila Thangaratinam, Ewelina Rogozińska, Helena J. Teede

**Affiliations:** 1 Monash Centre for Health Research and Implementation, School of Public Health and Preventive Medicine, Monash University, Melbourne, Victoria, Australia; 2 Diabetes and Endocrine Unit, Monash Health, Melbourne, Victoria, Australia; 3 World Health Organization Collaborating Centre for Global Women’s Health, Institute of Metabolism and Systems Research, University of Birmingham, Birmingham, United Kingdom; 4 Birmingham Women’s and Children’s National Health Service Foundation Trust, Birmingham, United Kingdom; 5 Medical Research Council Clinical Trials Unit at University College London, Institute of Clinical Trials and Methodology, London, United Kingdom

## Abstract

**Importance:**

Randomized clinical trials have found that antenatal lifestyle interventions optimize gestational weight gain (GWG) and pregnancy outcomes. However, key components of successful interventions for implementation have not been systematically identified.

**Objective:**

To evaluate intervention components using the Template for Intervention Description and Replication (TIDieR) framework to inform implementation of antenatal lifestyle interventions in routine antenatal care.

**Data Sources:**

Included studies were drawn from a recently published systematic review on the efficacy of antenatal lifestyle interventions for optimizing GWG. The Cochrane Database of Systematic Reviews, Database of Abstracts of Reviews of Effects, Cochrane Central Register of Controlled Trials, Health Technology Assessment Database, MEDLINE, and Embase were searched from January 1990 to May 2020.

**Study Selection:**

Randomized clinical trials examining efficacy of antenatal lifestyle interventions in optimizing GWG were included.

**Data Extraction and Synthesis:**

Random effects meta-analyses were used to evaluate the association of intervention characteristics with efficacy of antenatal lifestyle interventions in optimizing GWG. The results are reported according to the Preferred Reporting Items for Systematic Reviews and Meta-analyses reporting guideline. Data extraction was performed by 2 independent reviewers.

**Main Outcomes and Measures:**

The main outcome was mean GWG. Measures included characteristics of antenatal lifestyle interventions comprising domains related to theoretical framework, material, procedure, facilitator (allied health staff, medical staff, or researcher), delivery format (individual or group), mode, location, gestational age at commencement (<20 wk or ≥20 wk), number of sessions (low [1-5 sessions], moderate [6-20 sessions], and high [≥21 sessions]), duration (low [1-12 wk], moderate [13-20 wk], and high [≥21 wk]), tailoring, attrition, and adherence. For all mean differences (MDs), the reference group was the control group (ie, usual care).

**Results:**

Overall, 99 studies with 34 546 pregnant individuals were included with differential effective intervention components found according to intervention type. Broadly, interventions delivered by an allied health professional were associated with a greater decrease in GWG compared with those delivered by other facilitators (MD, −1.36 kg; 95% CI, −1.71 to −1.02 kg; *P* < .001). Compared with corresponding subgroups, dietary interventions with an individual delivery format (MD, −3.91 kg; 95% CI −5.82 to −2.01 kg; *P* = .002) and moderate number of sessions (MD, −4.35 kg; 95% CI −5.80 to −2.89 kg; *P* < .001) were associated with the greatest decrease in GWG. Physical activity and mixed behavioral interventions had attenuated associations with GWG. These interventions may benefit from an earlier commencement and a longer duration for more effective optimization of GWG.

**Conclusions and Relevance:**

These findings suggest that pragmatic research may be needed to test and evaluate effective intervention components to inform implementation of interventions in routine antenatal care for broad public health benefit.

## Key Points

**Question:**

What are the efficacious components of antenatal lifestyle interventions to inform implementation in antenatal care settings?

**Findings:**

In this meta-analysis of 99 randomized clinical trials of antenatal lifestyle interventions among 34 546 pregnant individuals, intervention delivery by an allied health professional was associated with optimized gestational weight gain (GWG). Among dietary interventions, previously found to be associated with a greater decrease in GWG, those with individual delivery format and moderate intensity were associated with the greatest decrease in GWG, while physical activity and mixed behavioral interventions may benefit with earlier commencement and a longer duration for effective associations with decreased GWG.

**Meaning:**

These findings provide insight to characteristics of efficacious interventions, as well as those that may be considered adaptable according to contextual needs and available resources.

## Teaser

This meta-analysis investigates the association of components of antenatal lifestyle interventions with optimized gestational weight gain among pregnant individuals.

## Introduction

Accelerated weight gain before, during, and after pregnancy is prevalent, with approximately 70% of female adults in the US^1^ experiencing overweight or obesity. Pregnancy presents a critical risk, with half of all pregnant individuals exceeding recommendations for gestational weight gain (GWG),^2^ with associated increased adverse risk of maternal and neonatal sequale,^3^ including obesity development.^4^ Therefore, optimizing GWG during pregnancy with lifestyle intervention has been advocated as a public health strategy to reduce maternal weight accretion, as emphasized by the US Preventive Services Task Force.^5^ In our review^6^ of 117 randomized clinical trials (RCTs), antenatal lifestyle intervention was associated with decreased GWG and reduced risk of gestational diabetes and total adverse maternal outcomes compared with routine care. Differential effects were noted by intervention type, with dietary intervention associated with the greatest benefits for optimizing GWG compared with physical activity, diet with physical activity, or mixed interventions overall. With demonstrated cost-effectiveness,^7,8^ pragmatic implementation of effective interventions in routine care remains a vital next step to leverage investment in the evidence generated to date.^9^ However, in the context of highly heterogeneous intervention design, limited guidance exists on what interventions should be implemented and how, curtailing effective translation into antenatal care settings.^10^

Frameworks to guide implementation of programs and interventions into practice, such as the Consolidated Framework Implementation Research (CFIR), emphasize the identification of core intervention characteristics as part of this process. Alongside the intervention to be implemented, the CFIR comprises 5 domains, including the outer setting (ie, policy, guidelines, and population needs), inner setting (ie, organizational structure, culture, and readiness to change), individuals who influence implementation, and iterative processes, including executing and evaluating implementation activities.^11^ The framework proposes that an intervention retains core, or essential, characteristics that are fundamental to intervention efficacy and peripheral, or adaptable, characteristics informed by inner and outer settings and individuals within the intervention setting.^11^ This presents a pragmatic approach in defining what intervention components are essential for efficacy compared with those that can be adapted to best meet the context, health system, resource setting, and population needs during implementation design.^11^

To date, published systematic reviews in the field are limited in providing understanding of effective components beyond classification of intervention type (eg, diet, physical activity, mixed, or behavioral). Consequently, implementation research remains stalled without detailed knowledge of optimal components that comprise an intervention, including but not limited to delivery mode, format, intensity, facilitator type, and training.^12,13^ In a secondary analysis of our 2022 systematic review reporting on the associations of lifestyle interventions with efficacy in optimizing GWG,^6^ this meta-analysis aims to elucidate and describe components of antenatal lifestyle interventions that are associated with optimized GWG within published RCTs, providing critical and pragmatic information for implementation of trials in antenatal care settings.

## Methods

### Search Strategy and Study Selection

This meta-analysis and the original systematic review and meta-analysis^6^ were reported according to the Preferred Reporting Items for Systematic Reviews and Meta-analyses (PRISMA) reporting guideline. This is a secondary analysis of a recent systematic review and meta-analysis^6^ to expand on the association of intervention components with optimization of GWG according to the Template for Intervention Description and Replication (TIDieR) framework.^14^ The TIDieR framework is an extension of the Consolidated Standards of Reporting Trials (CONSORT) and SPIRIT reporting templates and is designed to enhance replicability of interventions by including domains of what (eg, resources, materials, and procedure), who (eg, the facilitator), how (eg, the delivery format), where (eg, the setting), when (eg, intervention commencement), and how much (eg, frequency and intensity), along with tailoring and factors associated with fidelity.^14^

Systematic review methods have been reported in detail elsewhere.^6,15,16,17^ In brief, we searched the Cochrane Database of Systematic Reviews, Database of Abstracts of Reviews of Effects, Cochrane Central Register of Controlled Trials, Health Technology Assessment Database, MEDLINE, and Embase up to May 6, 2020, with no language restrictions. Eligible studies were identified as RCTs of antenatal diet, physical activity, or mixed interventions in pregnant individuals that reported mean GWG per group. Studies were ineligible if they recruited individuals with multiple pregnancies or preexisting conditions (eg, gestational diabetes); involved non–lifestyle interventions (ie, GWG monitoring only), or were published prior to 1990. Eligibility of the studies was assessed by 2 reviewers (including M.B.K.) independently, and discrepancies were resolved by a third reviewer (H.J.T.).

### Data Extraction

Data extraction on general study characteristics (eg, author, year of publication, country, sample size, mean body mass index [BMI; calculated as weight in kilograms divided by height in meters squared] for total included population, and mean [SD] of GWG per group) was performed by 2 reviewers (including M.B.K.). Countries where interventions were conducted were classified according to United Nations definitions.^18^ Details of intervention classification were reported previously.^6^ In brief, diet interventions were classified as those using specified dietary targets (self-directed or facilitator led [researcher, instructor, trainer, or dietitian]) with or without monitoring (logs, recalls, or diaries) and with or without supply of food. Physical activity interventions were classified as those conducted in controlled conditions (research facility, gym, or classes) or a minority that were structured but self-led (activity targets and equipment provided). Diet with physical activity required at least 1 structured component, and mixed interventions were classified as those not meeting the listed criteria for structured interventions and that instead included a combination of lifestyle advice, with or without weight monitoring, those that included behavioral strategies alone, or those in which structured diet and physical activity components were not adequately described.^6^

Descriptions of intervention characteristics according to the TIDieR framework^14^ are presented in Table 1. Characteristics included the theoretical framework underpinning the intervention, resources provided to the intervention group (eg, pamphlets, manual, handouts, and GWG charts), intervention facilitators (eg, allied health professional, medical staff, or researcher), intervention training provided to the facilitator delivering the intervention, mode (eg, face to face or remote) and format (eg, group or individual) of intervention delivery, setting of intervention delivery (eg, clinical setting or exercise facility), number (low [1-5 sessions], moderate [6-20 sessions], or high [≥21 sessions]) and duration of delivered sessions, gestational age at commencement (<20 wk or ≥20 wk) and completion of interventions, intervention duration in weeks (low [1-12 wk], moderate [13-20 wk], and high [≥21 wk]), tailoring applied to the intervention (eg, personalized to participant), and intervention adherence and attrition.

**Table 1.** Description of Intervention Characteristics According to the TIDieR Framework

TIDieR checklist item	Description	Variable adapted from TIDieR checklist
Why (rationale/theory)	Rationale, theory, or goal of elements essential to the intervention	Behavioral theory: must state a behavioral change theory or approach used to support design or delivery of the intervention. Examples could include social cognitive theory and motivational interviewing Yes No or not reported
What (materials)	Physical or informational materials used in the intervention	Resources provided to participants: Self-monitoring tool: GWG charts and self-monitoring tools (pedometers and exercise diary) Other resource, including written or electronic brochure, handbook, manual, article link, or website Combination: self-monitoring tool with other resource None
What (procedure)	Procedures, activities, or processes used in the intervention	Type of intervention: Diet Physical activity Diet and physical activity Mixed
Who provided	Intervention facilitator and their expertise, background, and any specific training given	Intervention facilitator: Allied health staff: defined as nonmedical health trained staff, including physiotherapist; exercise physiologist, scientist, or trainer; gym, aerobics, or fitness instructor; kinesiologist; dietitian; food technologist; nutritionist; and community health worker Medical staff: medical or nursing trained facilitator, including obstetrician, gynecologist, clinician, doctor, midwife, and nurse Other: category not fitting allied health staff or medical staff, including researcher, health coach, peer facilitator, and not reported eHealth: coded as not applicable given that facilitator not provided Prior training: Was intervention-specific training received prior to delivering the intervention? (This does not refer to the educational or professional background of this person.) Yes No or not reported
How	Modes of delivery (face to face or remote) of the intervention and whether provided individually or in a group	Mode of intervention delivery: Face to face Remote Face to face and remote Intervention format: Where there was a combination of individual and group delivery, the format that most sessions were delivered by was considered Individual session Group session
Where	Physical location where the intervention was carried out. Note: this is independent of where recruitment took place, with most trials recruiting from antenatal care clinics	Location: Exercise center Hospital or antenatal clinic Other: not exercise center, hospital, or antenatal clinic, including home-based session and research center
When and how much	No. of planned intervention sessions delivered to the participant, not inclusive of ongoing or optional support or provision of resources The week when the first session was delivered Length of sessions in minutes No. of weeks between first and last weeks of intervention delivery	No. sessions: Low: 1-5 Moderate: 6-20 High: ≥21 Gestational age at commencement, w Early pregnancy: <20 Late pregnancy: ≥20 Length of sessions, min: Where a range was reported, the lower limit was considered (as the length of session provided to all participants for sure). Where different lengths were reported for each format or method type, the mean was considered Low: 1-30 Moderate: 31-60 High: ≥61 Not reported Intervention duration, wk Low: 1-12 Moderate: 13-20 High: ≥21 Not able to calculate: first or last week of intervention delivery was not reported
Tailoring	If the intervention was planned to be personalized, titrated, or adapted, then describe what, why, when, and how	Tailoring: Was the intervention planned to be personalized or adapted for the participant? Yes No or not reported
How well actual	Participant attrition and adherence. Attrition: dropout rate of the intervention reported at conclusion of the study excluding medical indications. Adherence: adherence to the delivery of the intervention in relation to attendance of sessions. This will be entered as a numerical character sourced from the referenced research study and expressed as a % value	Attrition, % Low: <15 High: ≥15 Not reported Compliance, % Low: <75 High: ≥75 Not reported
GWG	GWG as reported in the study	Intervention group: GWG mean_i (kg): mean GWG in intervention group GWG sd_i: GWG in intervention group: SD total_i (n): No. participants in intervention group Control group: GWG mean_c (kg): mean GWG in control group GWG sd_c: GWG in control group - SD total_c (n): No. participants in control group
Intervention vs control	Intervention vs control: was the GWG statistically significant as reported in the study?	Significant: GWG in intervention vs control comparison statistically significant as reported in study Not significant: GWG intervention vs control comparison not statistically significant as reported in study
Ongoing support	Direct support or contact provided independent to intervention sessions as part of the intervention	Direct ongoing support or contact provided independent to intervention sessions as part of the intervention. Examples include text message, email, telephone, and mail Yes No

Abbreviations: GWG, gestational weight gain; TIDieR, Template for Intervention Description and Replication.

### Statistical Analysis

The primary outcome was mean difference (MD) with 95% CI of GWG using the intention-to-treat principle. We assessed the association of TIDieR components with efficacy of lifestyle interventions overall and by intervention type using subgroup random effects meta-analysis of effect statistics to calculate summary effect estimates and 95% CIs (applying DerSimonian and Laird random effects models using the metan Stata command^19^). For all MDs, the reference group was the control group (ie, usual care). Heterogeneity was assessed using the *I*^2^ statistic, with *I*^2^ > 50% indicating substantial heterogeneity.^20^ To account for 15 multiple comparisons, which increase the risk of type I errors, we applied a Bonferroni correction and the statistical significance was set at a 2-sided *P* < .003. Given this more stringent significance level when making comparisons between the 15 Tidier factors examined, *P* values are reported as well as 95% CIs. Statistical analyses were conducted using Stata statistical software version 16 (StataCorp).

## Results

Of 117 studies included in our systematic review,^6^ 99 studies^21,22,23,24,25,26,27,28,29,30,31,32,33,34,35,36,37,38,39,40,41,42,43,44,45,46,47,48,49,50,51,52,53,54,55,56,57,58,59,60,61,62,63,64,65,66,67,68,69,70,71,72,73,74,75,76,77,78,79,80,81,82,83,84,85,86,87,88,89,90,91,92,93,94,95,96,97,98,99,100,101,102,103,104,105,106,107,108,109,110,111,112,113,114,115,116,117,118,119^ reported GWG data and were included in this TIDieR meta-analysis (eFigure in Supplement 1),^6,120^ with general characteristics of studies previously reported.^6^ Of 34 546 pregnant individuals recruited, the mean (SD) baseline BMI ranged from 20.6 (2.5)^21^ to 38.6 (6.1).^22^ Most studies in this analysis were conducted in high-income countries (81 studies [81.8%]).^22,23,24,25,26,32,33,35,36,38,39,40,41,42,43,44,45,46,47,48,49,50,51,52,53,54,55,56,57,58,60,62,63,64,66,67,70,71,72,73,74,75,76,77,78,79,80,81,83,84,85,86,88,89,90,91,92,93,94,96,97,98,99,100,101,102,103,104,105,106,107,108,109,110,111,113,115,116,117,118,119^ As previously reported, lifestyle interventions were associated with an MD in GWG by 1.15 kg (95% CI, −1.40 to −0.91 kg) compared with control groups, with all intervention types found to be efficacious.^6^

### Overall Intervention Characteristics and Association With GWG

Table 2 summarizes intervention characteristics according to the TIDieR framework,^21,22,23,24,25,26,27,28,29,30,31,32,33,34,35,36,37,38,39,40,41,42,43,44,45,46,47,48,49,50,51,52,53,54,55,56,57,58,59,60,61,62,63,64,65,66,67,68,69,70,71,72,73,74,75,76,77,78,79,80,81,82,83,84,85,86,87,88,89,90,91,92,93,94,95,96,97,98,99,100,101,102,103,104,105,106,107,108,109,110,111,112,113,114,115,116,117,118,119^ and Table 3 summarizes the association of intervention characteristics with efficacy in decreased GWG (see eTables 1-4 in Supplement 1 for subgroup analyses by intervention type). Overall, most interventions were delivered in a group format (51 studies [51.5%])^23,24,25,26,27,28,29,30,31,32,33,34,35,36,37,38,39,40,41,42,43,44,45,46,47,48,49,50,51,52,53,54,55,56,57,58,59,60,61,62,63,64,65,66,67,68,69,70,71,72,73^ using a face-to-face delivery mode (84 studies [84.8%]),^21,22,23,24,25,26,27,28,29,30,31,32,33,34,35,36,37,38,39,40,41,42,43,44,45,46,47,49,50,51,52,53,55,56,57,58,59,60,61,62,63,65,66,67,68,69,70,71,72,73,74,75,76,77,78,79,80,81,82,83,84,85,86,87,88,89,90,91,92,93,94,95,96,97,98,99,100,101,102,103,104,105,106,107^ with no distinguishable differences across formats or modes in GWG outcome. Most studies (67 studies [67.7%]) did not train intervention facilitators or did not report training.^22,24,25,26,28,29,30,31,32,34,35,36,38,39,40,42,43,44,46,47,48,49,50,51,53,55,56,58,59,60,62,65,66,67,71,72,73,74,75,78,79,80,81,83,84,85,88,89,91,92,93,94,96,97,98,99,101,107,108,109,110,111,112,113,114,115,116^ Efficacy in GWG outcomes differed significantly by intervention facilitator type (*P* < .001), with allied health staff being the most efficacious (MD, −1.36 kg; 95%CI, −1.71 to −1.02 kg)^22,25,26,27,30,31,32,33,35,36,37,38,39,40,41,43,44,46,47,48,49,51,53,55,56,58,60,61,63,64,65,66,67,69,70,72,73,74,75,76,77,80,81,83,84,85,87,88,89,90,91,92,93,97,98,99,101,102,103,104,108,114,116,117^ and no facilitator (ie, remote delivery) being nonefficacious (MD, −0.25 kg; 95% CI, −0.98 to 0.48 kg).^107,109,111,113,115^ Most interventions were delivered in early pregnancy (67 studies [67.7%])^21,22,24,26,31,32,33,35,38,39,42,43,44,45,46,47,50,53,54,55,56,57,58,59,60,62,63,65,66,67,68,69,70,71,72,75,76,77,78,79,82,83,85,86,88,89,91,92,93,94,95,98,99,100,101,102,103,104,105,106,111,112,113,114,116,117,118^ and in a clinical setting (68 studies [68.7%]).^21,23,24,26,28,29,32,33,34,35,37,38,39,41,42,43,44,45,47,51,53,54,55,56,57,58,59,60,62,63,66,67,68,69,70,71,72,75,76,77,78,80,82,84,85,86,87,88,89,90,91,93,94,95,96,98,99,102,103,105,106,108,109,112,114,116,118,119^ Most studies involved interventions with a high (40 studies [40.4%])^24,25,27,28,29,30,31,32,34,35,36,38,39,41,42,44,46,47,48,49,51,53,55,59,61,62,64,65,66,70,71,72,73,79,92,95,104,113^ or low (33 studies [33.3%])^21,22,26,33,43,45,50,57,60,67,68,69,75,77,78,82,83,86,88,90,91,93,96,97,98,99,102,106,107,109,112,116,119^ number of sessions, low (24 studies [24.2%])^21,24,26,34,48,49,59,63,64,82,84,86,96,97,99,101,103,105,106,109,110,114,117,119^ to moderate (47 studies [47.5%]),^22,23,25,27,28,29,30,31,32,33,35,36,37,38,39,40,41,42,44,46,47,51,53,54,55,56,58,60,61,62,65,66,70,71,72,73,76,79,81,88,91,92,95,98,100,104,116^ length, and moderate (36 studies [36.4%])^27,33,43,45,49,50,51,56,57,61,62,64,69,73,75,81,82,83,86,90,96,97,100,101,102,104,106,107,110,111,112,113,115,116,117,119^ to high (32 studies [32.3%])^26,31,32,35,38,39,42,44,46,47,53,55,58,59,63,65,66,71,72,78,85,89,91,93,94,98,99,103,105,118^ duration. There were 23 studies (23.2%) reporting provision of ongoing support.^21,22,23,49,52,54,63,64,67,74,83,93,95,96,97,99,102,103,112,115,116,118,119^ Attrition was low in most studies (56 studies [56.6%]).^21,22,32,33,35,36,37,38,39,40,43,44,46,48,50,53,54,55,57,61,62,64,65,66,67,68,70,72,73,75,78,81,84,86,88,89,90,92,93,94,95,99,100,101,102,103,104,106,107,108,109,111,112,113,114,115,117^ However, adherence was not commonly reported (50 studies [50.5%]).^21,25,26,27,30,31,33,34,37,40,41,43,49,53,57,58,63,67,68,69,74,75,78,80,82,83,84,85,86,87,88,89,90,91,94,95,98,99,100,102,103,105,107,108,109,110,111,112,114,119^ The association of intervention session number, length of sessions, duration of intervention, provision of ongoing support or resources, tailoring, adherence, and attrition with reduction in GWG could not be delineated.

**Table 2.** Intervention Characteristics of Lifestyle Interventions in Pregnancy

Study	Country (No.) [mean BMI]	Intervention type; theory; resource	Intervention format; delivery mode	Intervention facilitator; location	Intervention duration, wk	No. sessions (min/session)	Tailoring; adherence
Kihlstrand et al,^23^ 1999	Sweden (241) [NR]	Physical activity; NA theory; none	Group; face to face	Medical staff (trained); clinical setting	20	20 (60)	Not tailored; 0.552
Clapp et al,^24^ 2000	US (46) [NA]	Physical activity; NA theory; none	Group; face to face	Others; clinical setting	32	96 (20)	Not tailored; 0.45
Marquez-Sterling et al,^25^ 2000	US (15) [23.7]	Physical activity; NA theory; none	Group; face to face	Allied health staff; exercise center	15	45 (60)	Not tailored; NA
Blackwell et al,^26^ 2002	US (46) [NA]	Diet; NA theory; none	Group; face to face	Allied health staff; clinical setting	24	3 (20)	Not tailored; NA
Briley et al,^74^ 2002	US (20) [24]	Mixed; NA theory; other resources	Individual; face to face	Allied health staff; home-based session	9	6 (NA)	Tailored; NA
Polley et al,^108^ 2002	US (110) [27.7]	Mixed; NA theory; combination	Individual; face to face and remote	Allied health staff; clinical setting	19	NA (NA)	Tailored; NA
Prevedel et al,^27^ 2003	Brazil (39) [24.7]	Physical activity; NA theory; none	Group; face to face	Allied health staff (trained); exercise center	19	57 (60)	Not tailored; NA
Garshasbi et al,^28^ 2005	Iran (266) [25.8]	Physical activity; NA theory; none	Group; face to face	Medical staff; clinical setting	12	36 (60)	Not tailored; 0.916
Khoury et al,^75^ 2005	Norway (289) [24.3]	Diet; NA theory; other resources	Individual; face to face	Allied health staff; clinical setting	18	4 (NA)	Tailored; NA
Santos et al,^29^ 2005	Brazil (90) [27.8]	Physical activity; NA theory; none	Group; face to face	Others; clinical setting	12	36 (60)	Not tailored; 0.4
Sedaghati et al,^30^ 2007	Iran (90) [24.2]	Physical activity; NA theory; none	Group; face to face	Allied health staff; research center	8	24 (45)	Not tailored; NA
Baciuk et al,^31^ 2008	Brazil (70) [NA]	Physical activity; NA theory; other resources	Group; face to face	Allied health staff; exercise center	21	60 (50)	Not tailored; NA
Barakat et al,^32^ 2008	Spain (140) [23.8]	Physical activity; NA theory; none	Group; face to face	Allied health staff; antenatal clinic	26	78 (35)	Tailored; 0.90
Wolff et al,^76^ 2008	Denmark (59) [34.9]	Diet; NA theory; none	Individual; face to face	Allied health staff; clinical setting	19	10 (60)	Tailored; 0.913
Asbee et al,^77^ 2009	US (100) [26.1]	Diet with physical activity; NA theory; none	Individual; face to face	Allied health staff (trained); clinical setting	1	1 (NA)	Tailored; 0.614
Jeffries et al,^78^ 2009	Australia (282) [25.7]	Mixed; NA theory; self-monitoring tool	Individual; face to face	Medical staff; clinical setting	22	2 (NA)	Not tailored; NA
Ong et al,^79^ 2009	Australia (12) [36.0]	Physical activity; NA theory; other resources	Individual; face to face	Others; home-based session	10	30 (45)	Not tailored; 0.94
Thornton et al,^80^ 2009	US (232) [37.8]	Diet; NA theory; self-monitoring tool	Individual; face to face	Allied health staff; clinical setting	12	NA (NA)	Not tailored; NA
Guelinckx et al,^33^ 2010	Belgium (195) [33.6]	Mixed; theory based; other resources	Group; face to face	Allied health staff; clinical setting	17	3 (60)	Tailored; NA
Hopkins et al,^81^ 2010	New Zealand (84) [25.5]	Physical activity; NA theory; combination	Individual; face to face	Allied health staff; home-based session	16	8 (40)	Tailored; 0.75
Khaledan et al,^34^ 2010	Iran (39) [28.3]	Physical activity; NA theory; none	Group; face to face	Others; clinical setting	8	24 (30-45)	Not tailored; NA
Barakat et al,^35^ 2011	Spain (67) [NA]	Physical activity; NA theory; none	Group; face to face	Allied health staff; clinical setting	29	85 (35-45)	Tailored; 0.90
Haakstad et al,^36^ 2011	Norway (101) [25.3]	Physical activity; NA theory; self-monitoring tool	Group; face to face	Allied health staff; exercise center	12	24 (60)	Tailored; 0.71
Huang et al,^82^ 2011	Taiwan (189) [21.0]	Mixed; NA theory; combination	Individual; face to face	Medical staff (trained); clinical setting	20	3 (30-40)	Tailored; NA
Jackson et al,^109^ 2011	US (287) [27]	Mixed; theory based; other resources	Individual; remote	eHealth; clinical setting	4	NA (NA)	Tailored; NA
Nascimento et al,^37^ 2011	Brazil (80) [36.9]	Physical activity; NA theory; self-monitoring tool	Group; face to face	Allied health staff; clinical setting	12	12 (40)	Tailored; NA
Phelan et al,^83^ 2011	US (393) [27.4]	Mixed; theory based; combination	Individual; face to face	Allied health staff; research center	14	1 (NA)	Tailored; NA
Quinlivan et al,^84^ 2011	Australia (124) [NA]	Diet; NA theory; none	Individual; face to face	Allied health staff; clinical setting	NA	NA (5)	Tailored; NA
Barakat et al,^39^ 2012	Spain (290) [22.9]	Physical activity; NA theory; none	Group; face to face	Allied health staff; clinical setting	29	87 (40-45)	Tailored; 0.87
Barakat et al,^38^ 2012a	Spain (83) [24.4]	Physical activity; NA theory; none	Group; face to face	Allied health staff; clinical setting	29	87 (35-45)	Tailored; 0.85
Hui et al,^40^ 2012	Canada (183) [NA]	Diet with physical activity; NA theory; combination	Individual (2 diet) and group (10 exercise; face to face)	Allied health staff; exercise center	10	12 (45)	Tailored; 0.87
Korpi-Hyövälti et al,^85^ 2012	Finland (54) [26.4]	Diet; NA theory; other resources	Individual; face to face	Allied health staff; clinical setting	24	6 (NA)	Tailored; NA
Oostdam et al,^41^ 2012	Netherlands (105) [35.6]	Physical activity; NA theory; none	Group; face to face	Allied health staff (trained); clinical setting	25	50 (60)	Tailored; 0.111
Price et al,^42^ 2012	US (62) [27.7]	Physical activity; NA theory; none	Group; face to face	Medical staff; clinical setting	22	66 (45-60)	Tailored; 0.930
Walsh et al,^43^ 2012	Ireland (759) [27.1]	Diet; NA theory; other resources	Group; face to face	Allied health staff; clinical setting	16	3 (120)	Not tailored; NA
Althuizen et al,^86^ 2013	Netherlands (269) [27.6]	Mixed; theory based; other resources	Individual; face to face	Others; clinical setting	17	4 (30)	Tailored; 0.67
Barakat et al,^44^ 2013	Spain (279) [23.9]	Physical activity; NA theory; none	Group; face to face	Allied health staff; clinical setting	26	78 (50-55)	Tailored; 0.95
Bogaerts et al,^45^ 2013	Belgium (197) [34.7]	Mixed; theory based; other resources	Group; face to face	Medical staff (trained); clinical setting	20	4 (90-120)	Tailored; 0.79
Deveer et al,^87^ 2013	Turkey (100) [28.6]	Diet; NA theory; none	Individual; face to face	Allied health staff; clinical setting	12	8 (NA)	Tailored; NA
Harrison et al,^88^ 2013	Australia (238) [31.4]	Mixed; theory based; combination	Individual; face to face	Allied health staff; clinical setting	12	4 (60)	Tailored; NA
Ruiz et al,^46^ 2013	Spain (927) [NA]	Physical activity; NA theory; none	Group; face to face	Allied health staff; exercise center	29	87 (50-55)	Tailored; 0.97
Barakat et al,^47^ 2014	Spain (200) [23.9]	Physical activity; NA theory; none	Group; face to face	Allied health staff; clinical setting	26	78 (55-60)	Tailored; 0.95
Di Carlo et al,^89^ 2014	Italy (120) [25.8]	Diet; NA; other resources	Individual; face to face	Allied health staff; clinical setting	24	6 (NA)	Tailored; NA
Dodd et al,^90^ 2014	Australia (2199) [32.49]	Mixed; theory based; combination	Individual; face to face and remote	Allied health staff (trained); clinical setting	16	3 (NA)	Tailored; 0.77
Hui et al,^48^ 2014	Canada (113) [NA]	Diet with physical activity; NA theory; combination	Individual and group; face to face and remote	Allied health staff; exercise center	10	30-80 (45)	Tailored; 1
Ko et al,^49^ 2014	US (1196) [25.7]	Physical activity; theory based; other resources	Group; face to face	Allied health staff; exercise center	16	52 (30)	Tailored; NA
Kong et al,^50^ 2014	US (37) [30.7]	Physical activity; NA; combination	Group; face to face	Others; home-based session	20	1 (NA)	Not tailored; 1.0
Petrella et al,^91^ 2014	Italy (61) [33.8]	Diet with physical activity; NA theory; self-monitoring tool	Individual; face to face	Allied health staff; clinical setting	24	5 (60)	Tailored; NA
Vesco et al,^51^ 2014	US (114) [36.7]	Diet with physical activity; theory based; self-monitoring tool	Individual and group; face to face	Allied health staff; clinical setting	13	18 (45- 90)	Tailored; 0.816
Bisson et al,^92^ 2015	Canada (45) [34.75]	Physical activity; NA theory; none	Individual; face to face	Allied health staff; exercise center	12	36 (60)	Tailored; 0.5135
Dekker et al,^93^ 2015	Australia (35) [36.8]	Physical activity; NA theory; other resources	Individual; face to face	Allied health staff; clinical setting	24	4 (NA)	Tailored; 1.0
Gesell et al,^52^ 2015	US (87) [NA]	Diet with physical activity; theory based; none	Group; face to face	Others (trained); exercise center	12	12 (90)	Tailored; 0.333
Hawkins et al,^118^ 2015	US (68) [NA]	Mixed; theory based; combination	Individual; face to face	Others (trained); clinical setting	22	6 (NA)	Tailored; 0.66
Jing et al,^21^ 2015	China (221) [20.6]	Mixed; theory based; other resources	Individual; face to face	Others (trained); clinical setting	12	3 (20)	Tailored; NA
Perales et al,^53^ 2015	Spain (167) [NA]	Physical activity; NA theory; none	Group; face to face	Allied health staff; clinical setting	27	81 (60)	Tailored; NA
Poston et al,^54^ 2015	UK (1554) [36.3]	Mixed; theory based; combination	Individual and group; face to face and remote	Others (trained); clinical setting	8	8 (60)	Tailored; 0.875
Ronnberg et al,^94^ 2015	Sweden (374) [25.3]	Physical activity; NA theory; self-monitoring tool	Individual; face to face	Medical staff; clinical setting	24	NA (NA)	Tailored; NA
Aşcı et al,^95^ 2016	Turkey (90) [23.3]	Mixed; theory based; self-monitoring tool	Individual; face to face	Medical staff; clinical setting	5	3 (60)	Tailored; NA
Barakat et al,^55^ 2016	Spain (765) [23.5]	Physical activity; NA theory; none	Group; face to face	Allied health staff; clinical setting	27	81 (55)	Tailored; 0.80
Garnæs et al,^56^ 2016	Norway (74) [34.5]	Physical activity; theory based; self-monitoring tool	Individual and group; face to face	Allied health staff; clinical setting	18	37 (60)	Tailored; 0.5
Herring et al,^110^ 2016	US (56) [32.9]	Mixed; theory based; combination	Individual; remote	Others; home-based session	20	8 (15-20)	Tailored; 0.7
Koivusalo et al,^57^ 2016	Finland (269) [32.3]	Diet with physical activity; NA theory; self-monitoring tool	Individual and group; face to face	Allied health staff (trained); clinical setting	17	3 (120)	Tailored; NA
McCarthy et al,^96^ 2016	Australia (371) [30.3]	Mixed; NA theory; combination	Individual; face to face	Medical staff; clinical setting	16	1 (30)	Not tailored; 1.0
Perales et al,^58^ 2016	Spain (166) [NA]	Physical activity; NA theory; none	Group; face to face	Allied health staff; clinical setting	28	84 (55-60)	Tailored; NA
Seneviratne et al,^97^ 2016	New Zealand (75) [33.1]	Physical activity; NA theory; combination	Individual; face to face	Allied health staff; home-based session	15	1 (30)	Tailored; 0.33
Smith et al,^111^ 2016	US (45) [26.4]	Mixed; theory based; combination	Individual; remote	eHealth; home-based session	20	NA (NA)	Tailored; NA
Sun et al,^112^ 2016	China (66) [26.7]	Diet with physical activity; NA theory; none	Individual; face to face	Medical staff; clinical setting	16	5 (NA)	Tailored; NA
Wang et al,^59^ 2016	China (226) [26.8]	Physical activity; NA theory; none	Group; face to face	Others; hospital and antenatal clinic	24	72 (30)	Tailored; 0.73
Assaf-Balut et al,^60^ 2017	Spain (874) [23.9]	Diet; NA theory; none	Group; face to face	Allied health staff; clinical setting	1	1 (60)	Tailored; 1.0
Bruno et al,^98^ 2017	Italy (131) [34.2]	Diet with physical activity; NA theory; self-monitoring tool	Individual; face to face	Allied health staff; clinical setting	24	5 (60)	Tailored; 0.579
Chao et al,^117^ 2017	US (38) [31.2]	Diet with physical activity; theory based; self-monitoring tool	Individual; remote	Allied health staff (trained); others	20	20 (20)	Tailored; 0.625
da Silva et al,^61^ 2017	Brazil (594) [25.2]	Physical activity; NA theory; none	Group; face to face	Allied health staff (trained); exercise center	16	48 (60)	Tailored; 0.404
Daly et al,^62^ 2017	Ireland (76) [34.7]	Physical activity; NA theory; other resources	Group; face to face	Medical staff; clinical setting	19	57 (60)	Tailored; 0.789
Peaceman et al,^63^ 2017	US (280) [31]	Diet with physical activity; theory based; combination	Group; face to face	Allied health staff; clinical setting	21	6 (30)	Tailored; NA
Sagedal et al,^64^ 2017	Norway (600) [25.6]	Diet with physical activity; NA theory; other resources	Individual and group; face to face and remote	Allied health staff (trained); exercise center	16;	34 (20-60)	Tailored; 0.926
Sewell et al,^99^ 2017	UK (28) [NA]	Diet; theory based; other resources	Individual; face to face	Allied health staff; clinical setting	24	1 (15)	Tailored; NA
Simmons et al,^119^ 2017	UK (436) [36]	Mixed; theory based; combination	Individual; face to face and remote	Others (trained); clinical setting	15	5 (30-45)	Not tailored; NA
Willcox et al,^113^ 2017	Australia (91) [31]	Mixed; theory based; combination	Individual; remote	eHealth; others	19	NA (NA)	Tailored; 0.952
Bacchi et al,^65^ 2018	Argentina (111) [23.55]	Physical activity; NA theory; none	Group; face to face	Allied health staff; exercise center	27	85 (60)	Not tailored; 0.85
Barakat et al,^66^ 2018	Spain (325) [NA]	Physical activity; NA theory; none	Group; face to face	Allied health staff; clinical setting	27	81 (55-60)	Tailored; 0.80
Cahill et al,^100^ 2018	US (240) [32.4]	Mixed; theory based; none	Individual; face to face	Others (trained); others	20	10 (60)	Not tailored; NA
Chan et al,^114^ 2018	China (229) [23.6]	Diet with physical activity; NA theory; other resources	Individual; face to face and remote	Allied health staff; clinical setting	12	7 (20-30)	Tailored; 0.925
Kennelly et al,^67^ 2018	Ireland (535) [29.3]	Mixed; theory based; other resources	Individual and group; face to face	Allied health staff; clinical setting	22	3 (75)	Tailored; NA
Kiani Asiabar et al,^68^ 2018	Iran (150) [23.8]	Mixed; NA theory; other resources	Group; face to face	Medical staff (trained); clinical setting	1	2 (90)	Not tailored; NA
Olson et al,^115^ 2018	US (1689) [NA]	Mixed; theory based; combination	Individual; remote	eHealth; others	17	NA (NA)	Tailored; 0.461
Phelan et al,^101^ 2018	US (256) [32.5]	Diet with physical activity; theory based; combination	Individual; face to face	Allied health staff; research center	20	6 (20)	Tailored; 0.9
Al Wattar et al,^102^ 2019	UK (1252) [NA]	Diet; theory based; none	Individual and group; face to face	Allied health staff (trained); clinical setting	14	3 (NA)	Tailored; 0.74
Anleu et al,^69^ 2019	Chile (1002) [NA]	Diet; theory based; other resources	Group; face to face	Allied health staff (trained); clinical setting	13	3 (NA)	Tailored; NA
Barakat et al,^70^ 2019	Spain (520) [23.6]	Physical activity; NA theory; none	Group; face to face	Allied health staff (trained); clinical setting	28	84 (55-60)	Tailored; 0.8
Brik et al,^71^ 2019	Spain (120) [23.9]	Physical activity; NA theory; none	Group; face to face	Medical staff; clinical setting	22	66 (60)	Tailored; 0.70
Buckingham et al,^103^ 2019	US (56) [25]	Diet with physical activity; theory based; combination	Individual; face to face	Allied health staff (trained); clinical setting	22	6 (15-30)	Tailored; NA
Clark et al,^104^ 2019	US (42) [26.3]	Physical activity; NA theory; none	Individual; face to face	Allied health staff (trained); exercise center	20	60 (58-60)	Tailored; 0.79
Daley et al,^105^ 2019	UK (616) [26]	Mixed; theory based; self-monitoring tool	Individual; face to face	Medical staff (trained); clinical setting	24	8 (1-2)	Tailored; 0.51
Kunath et al,^106^ 2019	Germany (2261) [24.4]	Mixed; NA theory; combination	Individual; face to face	Medical staff (trained); clinical setting	14	3 (30-45)	Tailored; 0.85
Okesene-Gafa et al,^22^ 2019	New Zealand (230) [38.6]	Mixed; theory based; other resources	Individual; face to face	Allied health staff (NA); home-based setting	12	4 (30-60)	Tailored; 0.81
Pelaez et al,^72^ 2019	Spain (345) [23.7]	Physical activity; NA theory; none	Group; face to face	Allied health staff (NA); clinical setting	24	70 (60)	Not tailored; 0.80
Arthur et al,^107^ 2020	Australia (396) [27.5]	Mixed; NA theory; self-monitoring tool	Individual; face to face	Others; home-based setting	20	1 (NA)	Not tailored; NA
Ferrara et al,^116^ 2020	US (398) [29.4]	Diet with physical activity; theory based; combination	Individual; face to face and remote	Allied health staff; clinical setting	13	2 (55)	Not tailored; 0.81
Rodriquez-Blanque et al,^73^ 2020	Spain (162) [24.4]	Physical activity; NA theory; none	Group; face to face	Allied health staff; exercise center	17	51 (60)	Not tailored; 0.8

Abbreviations: BMI, body mass index (calculated as weight in kilograms divided by height in meters squared); NA, not available.

**Table 3.** Association of Lifestyle Intervention TIDieR Component Subgroups With GWGs

TIDieR intervention component	Studies (No.)	GWG, MD (95% CI), kg^a^	*I*^2^ (%)	*P* value for subgroup differences
Theory based				
Yes	33	−0.74 (−1.07 to −0.42)	73.7	.02
No or NA	66	−1.37 (−1.70 to −1.03)	79.5	
Resource				
Self-monitoring tool	14	−1.33 (−2.48 to −0.19)	87.5	.41
Other resource	22	−1.01 (−1.54 to −0.48)	74.4	
Combination	23	−0.81 (−1.17 to −0.45)	70.6	
None	40	−1.32 (−1.70 to −0.95)	75.2	
Format				
Individual	48	−1.34 (−1.74 to −0.93)	86.7	.22
Group	51	−1.03 (−1.31 to −0.75)	67.4	
Mode				
Face to face	84	−1.21 (−1.50 to −0.93)	78.5	.49
Remote	6	−0.31 (−1.46 to 0.84)	59.8	
Face to face and remote	9	−1.10 (−1.69 to −0.51)	57.4	
Facilitator				
Allied health staff	64	−1.36 (−1.71 to −1.02)	80.6	<.001
Medical staff	16	−0.85 (−1.41 to −0.28)	70.8	
Other	14	−0.91 (−1.39 to −0.43)	43.8	
NA	5	−0.25 (−0.98 to 0.48)	50.0	
Prior training				
Yes	32	−0.87 (−1.21 to −0.53)	66.1	.14
No or NA	67	−1.28 (−1.63 to −0.94)	88.0	
Location				
Hospital or antenatal clinic	68	−1.24 (−1.55 to −0.92)	80.5	.54
Exercise center	15	−0.99 (−1.59 to −0.40)	69.9	
Other	16	−0.93 (−1.58 to −0.29)	71.0	
Intervention commencement				
Early pregnancy	67	−1.09 (−1.35 to −0.83)	68.0	<.001
Late pregnancy	31	−1.13 (−1.62 to −0.64)	87.1	
NA	1	−6.80 (−8.63 to −4.97)		
Duration				
High	32	−1.26 (−1.57 to −0.95)	53.2	.47
Moderate	36	−0.91 (−1.25 to −0.56)	82.1	
Low	26	−1.28 (−1.91 to −0.65)	82.5	
Insufficient data to calculate	5	−2.60 (−6.19 to 0.98)	88.9	
No. of sessions				
High	40	−1.13 (−1.43 to −0.82)	60.9	.37
Moderate	20	−1.50 (−2.21 to −0.79)	71.9	
Low	33	−0.87 (−1.21 to −0.53)	68.3	
NA	6	−2.40 (−5.12 to 0.32)	96.8	
Ongoing support				
Yes	23	−0.91 (−1.33 to −0.49)	76.3	.22
No	76	−1.24 (−1.55 to −0.93)	80.2	
Length of session				
High	5	−1.42 (−2.42 to −0.43)	58.9	.72
Moderate	47	−1.09 (−1.39 to −0.79)	64.1	
Low	24	−0.94 (−1.47 to −0.41)	76.8	
NA	23	−1.55 (−2.17 to −0.94)	91.0	
Tailoring				
Tailored	74	−1.07 (−1.34 to −0.80)	78.1	.56
Not tailored or NA	25	−1.34 (−1.86 to −0.81)	83.5	
Compliance				
High	34	−1.18 (−1.55 to −0.82)	72.2	.88
Low	15	−0.99 (−1.70 to −0.28)	82.4	
Insufficient data to calculate	50	−1.17 (−1.58 to −0.76)	81.0	
Attrition				
High	15	−0.67 (−1.28 to −0.07)	59.6	.22
Low	57	−1.15 (−1.44 to −0.85)	86.0	
Insufficient data to calculate	27	−1.49 (−2.19 to −0.80)	84.1	

Abbreviations: GWG, gestational weight gain; MD, mean difference; NA: not available; TIDieR, Template for Intervention Description and Replication.

^a^ For all MDs, the reference group was the control group (ie, usual care).

### Association of Diet Intervention Characteristics With GWG

Of 13 dietary interventions (eTable 1 in Supplement 1),^26,43,60,69,75,76,80,84,85,87,89,99,102^ most were delivered in an individual format (9 studies [69.2%]),^75,76,80,84,85,87,89,99,102^ and all adopted a face-to-face delivery mode by allied health staff in a clinical setting.^26,43,60,69,75,76,80,84,85,87,89,99,102^ Individual delivery format (MD, −3.91 kg; 95% CI, −5.82 to −2.01 kg) was associated with a greater decrease in GWG compared with group format (MD, −0.23 kg; 95% CI, −1.28 to 0.82 kg; *P* = .002). Most studies comprised a low number of sessions (7 studies [53.8%]),^26,43,60,69,75,99,102^ with moderate (4 studies [30.8%])^43,69,75,102^ to high (4 studies [30.8%])^26,85,89,99^ intervention duration. Compared with corresponding subgroups, a moderate number of sessions (MD, −4.35 kg; 95% CI, −5.80 to −2.89 kg; *P* < .001) was associated with a greater decrease in GWG. Intervention tailoring, provision of resources, ongoing support, and attrition were not associated with GWG. Attrition was low (6 studies [46.2%])^43,75,84,89,99,102^ or not reported (6 studies [46.2%])^26,60,76,80,85,87^ in most studies. Most studies did not report adherence (11 studies [84.6%]).^26,43,69,75,80,84,85,87,89,99,102^

### Association Between Characteristics of Diet With Physical Activity Interventions and GWG

Of 16 diet with physical activity interventions (eTable 2 in Supplement 1), most were delivered by allied health staff (13 studies [81.3%]),^26,43,60,69,75,76,80,84,85,87,89,99,102^ using an individual format (9 studies [56.2%])^77,91,98,101,103,114,116,117^ and face-to-face mode (10 studies [62.5%]),^40,51,52,57,63,77,91,98,101,103^ with no significant differences across subgroups of facilitator, delivery format, or mode found. Interventions were mostly delivered in early pregnancy (11 studies [68.7%])^57,63,77,83,91,98,103,112,114,116,117^ and in a clinical setting (10 studies [62.5%]).^51,57,63,77,91,98,103,112,114,116^ Most studies involved interventions with a low (6 studies [37.5%])^57,77,91,98,112,116^ to moderate (7 studies [43.7%])^40,52,63,101,103,114,117^ number of sessions, low (7 studies [43.7%])^48,63,64,101,103,114,117^ to moderate (5 studies [31.2%])^40,51,91,98,116^ length, and low (5 studies [31.2%])^40,48,52,77,114^ to moderate (7 studies [43.7%])^51,57,64,101,112,116,117^ duration; 6 studies (37.5%) reported having ongoing support.^52,63,64,103,112,116^ In most studies, attrition was low (9 studies [56.2%])^40,48,57,64,101,103,112,114,117^ and adherence was not reported (8 studies [50.0%]).^40,57,63,91,98,103,112,114^ Intervention theoretical underpinning, delivery setting, number and length of sessions, duration of intervention, provision of ongoing support, tailoring, adherence, and attrition were not associated with differences in GWG reduction.

### Association of Physical Activity Intervention Characteristics and GWG

Of 42 physical activity interventions (eTable 3 in Supplement 1), most were delivered in a group format (35 studies [83.3%])^23,24,25,27,28,29,30,31,32,34,35,36,37,38,39,41,42,44,46,47,49,50,53,55,56,58,59,61,62,65,66,70,71,72,73^ by allied health staff (30 studies [71.4%])^25,27,30,31,32,35,36,37,38,39,41,44,46,47,49,53,55,56,58,61,65,66,70,72,73,81,92,93,97,104^ and all in face-to-face mode (42 studies [100%]),^23,24,25,27,28,29,30,31,32,34,35,36,37,38,39,41,42,44,46,47,49,53,55,56,58,59,61,62,65,66,70,71,72,73,79,81,92,93,94,97,104^ with no significant difference by intervention format, facilitator, or mode found. Interventions mostly commenced in early pregnancy (27 studies [64.3%])^24,31,32,35,38,39,42,44,46,47,50,53,55,56,58,59,62,65,66,70,71,72,79,92,93,94,104^ and occurred in a clinical setting (26 studies [61.9%]),^23,24,28,29,32,34,35,37,38,39,41,42,44,47,53,55,56,58,59,62,66,70,71,72,93,94^ with no association with efficacy. Most studies involved interventions with a high number of sessions (35 studies [83.3%]),^24,25,27,28,29,30,31,32,34,35,36,38,39,41,42,44,46,47,49,53,55,56,59,61,62,65,66,70,71,72,73,79,92,104^ moderate length (34 studies [81.0%]), ^23,25,27,28,29,30,31,32,35,36,37,38,39,41,42,44,46,47,53,55,58,66,70,79,81,92^ and high duration (20 studies [47.6%])^32,35,38,39,42,44,46,47,53,55,58,59,65,66,70,71,72,93,94^; 4 studies (9.5%)^23,49,93,97^ reported having ongoing support. In most studies, attrition was low (23 studies [54.8%])^32,35,36,37,38,39,44,46,50,53,55,61,62,65,66,70,72,73,81,92,93,94,104^ and reported adherence was high (21 studies [50.0%]).^28,32,35,38,39,42,44,46,47,50,55,62,65,66,70,72,73,79,81,93,104^ Theoretical underpinning, provision of resources or ongoing support, number and length of sessions, duration of intervention, tailoring, attrition, and adherence were not associated with GWG.

### Association of Mixed Intervention Characteristics and GWG

Of 28 mixed interventions (eTable 4 in Supplement 1), most were delivered in an individual format (23 studies [82.1%])^21,22,74,78,82,83,86,88,90,95,96,100,105,106,107,108,109,110,111,113,115,118,119^ and face-to-face mode (19 studies [67.9%]),^21,22,33,45,67,68,74,78,82,83,86,88,90,95,96,100,105,106,107^ with no significant difference in GWG reduction by subgroup. Intervention facilitator and prior training were not associated with the efficacy of interventions. Interventions were mostly delivered in early pregnancy (19 studies [67.9%])^21,22,33,45,54,67,68,78,82,83,86,88,95,100,105,106,111,113,118^ and in a clinical setting (19 studies [67.9%]).^21,22,33,45,54,67,68,78,82,86,88,90,95,105,106,108,109,111,118^ Most studies involved interventions with a low number of sessions (17 studies [60.7%])^21,22,33,45,67,68,78,82,83,86,88,90,106,107,109,119^ and moderate duration (15 studies [53.6%])^33,45,82,83,86,90,96,100,106,107,110,111,113,115,119^; 11 studies (39.3%)^21,22,54,67,74,83,95,96,115,118,119^ reported having ongoing support. Most interventions were tailored (22 studies [78.6%]).^21,22,33,45,54,67,74,82,83,86,88,90,95,105,106,108,109,110,111,113,115,118^ In most studies, attrition was low (19 studies [67.9%])^21,22,33,54,67,68,78,86,88,90,95,100,106,107,108,109,111,113,115^ but adherence was not reported (20 studies [71.4%]).^21,33,67,68,74,78,82,83,86,88,90,95,100,105,107,108,109,110,111,119^ Theoretical underpinning, commencement time and setting of intervention, number and length of sessions, duration of intervention, having ongoing support, tailoring, and attrition were not associated with a difference in GWG reduction; however, a high adherence level was associated with a higher efficacy (MD, −0.96 kg; 95%CI, −1.68 to −0.23 kg) compared with a low adherence (MD, <0.01 kg; 95% CI, −0.05 to 0.05 kg; *P* < .001).

## Discussion

The association of excess GWG with adverse maternal and neonatal outcomes has now been well established, as has the efficacy associated with lifestyle interventions, and population-based strategies to optimize GWG during pregnancy are recommended by the US Prevention Task Force.^5^ Despite supporting evidence for the cost-effectiveness of implementing interventions in antenatal care,^8^ little translation has been achieved to date, with a lack of clinical implementation-based research a critical remaining barrier. This is compounded by a lack of understanding of exactly what should be implemented and how. In this meta-analysis, we extended our recent systematic review^6^ to evaluate the association of intervention type with efficacy in reduced GWG. We also evaluated the potential association of specific pragmatic components of intervention design with efficacy, underpinned by the TIDieR framework, enabling exploration of efficacy of lifestyle interventions by who, what, when, where, and how much,^14^ which are important for informing implementation. In line with the CFIR, efficacious components have the potential to be considered core, or essential, to intervention design compared with less effective components that could be considered amenable according to contextual needs. Subgroup analysis by intervention type (diet, physical activity, diet with physical activity, and mixed interventions) found potential differences in characteristics by facilitator, delivery style, intensity level, and duration, which may provide significant insight to inform future implementation design of lifestyle interventions in antenatal care settings.

Our previous systematic review and meta-analysis^6^ built on several seminal reviews in the field to date, evaluating 117 RCTs to evaluate the association of antenatal lifestyle interventions with efficacy in optimized GWG and reduced risk of adverse maternal and neonatal outcomes. Differential effects were noted when analyzed by intervention type, with the greatest change in GWG found with diet (MD, −2.63 kg), followed by diet with physical activity (−1.35 kg), physical activity (−1.04 kg), and mixed interventions (−0.74 kg).^6^ Associated reductions in risk of adverse maternal and neonatal outcomes were demonstrated with diet interventions, while diet with physical activity and physical activity interventions were associated with reduced risk of maternal outcomes, and mixed interventions were associated with optimized GWG only.^6^ For a broad population health benefit to be realized, a vital next step is the pragmatic translation of interventions into routine care settings. Cost-effectiveness support implementation,^7,8^ with a 2022 study^8^ finding that for every A $1 (US $0.67) invested in implementation of structured diet and physical activity interventions, projected return was up to 5 times as high, with cost savings largely associated with reduced incidence of adverse outcomes. To enhance feasibility of implementation, a critical remaining gap is defining exactly what intervention components and strategies are associated with the greatest effectiveness in optimized GWG, nuanced to intervention type. This enables specificity in ensuring that the most effective characteristics are incorporated while enabling less efficacious characteristics to be modified according to local contextual factors related to resources, time, and cost. In turn, this may be associated with positive downstream cost-effectiveness and feasibility outcomes, particularly in resource-poor settings that may benefit greatly from population health initiatives.

Dietary interventions were associated with the greatest change in GWG, and on analysis, those delivered by allied health staff using a face-to-face mode and individual delivery format were associated with increased efficacy compared with group delivery. Interventions incorporating a moderate number of sessions (6-20 sessions) were also associated with optimized GWG, compared with a lower number of sessions (1-5 sessions). Dietary interventions need to consider pregnancy-specific barriers, including nausea, aversions, cravings and fatigue,^121^ widespread inadequate consumption of recommended fruit and vegetable servings, and increased availability of convenience foods, all of which may compromise diet quality.^122^ Given the complexity of individual barriers to optimal dietary composition, including environment, accessibility, sociodemographic factors, cultural practices, parity, and pregnancy-specific barriers, an individual delivery format may be more effective, as suggested by our findings, to maximize adherence to dietary advice or prescription that is difficult and complex to address in group-based formats. Peripheral and adaptable elements examined in this study, including intervention tailoring, behavioral and theoretical underpinning, session duration, and provision of ongoing support, were not found to be associated with a reduction in GWG. This is encouraging, suggesting the potential association of brief but frequent contact with a health professional with optimized GWG without the need for support between visits. In this study, dietary interventions were more prescriptive in nature, so it is perhaps not surprising that we found no evidence for the association of behavioral or theoretical underpinning with a change in GWG in dietary interventions. Key remaining questions include efficacy in clinical populations and delivery in routine care compared with highly select trial populations, as well as nuanced evidence on optimal time and intensity. Overall, our findings suggest that interventions delivered individually by an allied health professional and including 6 or more sessions may be considered as key components for dietary interventions.

Diet with physical activity, physical activity, and mixed interventions were associated with less effectiveness in optimizing GWG compared with dietary interventions. While no significant components were identified, for physical activity interventions, commencing in earlier pregnancy (ie, <20 weeks gestation), longer-duration interventions, and delivery by an allied health professional may be associated with more effectively optimized GWG. This is aligned to the well-accepted finding that physical activity interventions alone are associated with less reduction in weight compared with dietary interventions.^123^ These findings suggest that a longer intervention duration (ie, >20 weeks) commencing in early pregnancy may be advisable.

Mixed interventions were more likely to be focused on behavior change, commonly including goal setting, feedback, and monitoring and shaping knowledge. Behavior change is an iterative process, involving problem-solving and development of skills in self-management and self-efficacy, and an immediate association with weight change is less likely. Given that skills in behavior change require practice,^124^ commencing interventions earlier in pregnancy and over a longer duration may be considered relevant to GWG. In this study, we found that mixed interventions commencing at less than 20 weeks’ gestation and with longer duration and session length delivered in a group format were associated with lower GWG. Previous research found that a group format was associated with improved peer support.^125^ This may be particularly important during pregnancy to offer support directly related to the experience of pregnancy, which may not be available in a pregnant individual’s immediate social support network. In physical activity and mixed interventions, low attrition was associated with reduced GWG, which may have been related to the lower change in outcome associated with these intervention types compared with dietary interventions, which were associated with a greater change in GWG with fewer contact points.

### Strengths and Limitations

The strengths of this study include building on a robust systematic review and meta-analysis and intervention categorization spanning 30 years of research across 5 continents and involving 34 546 pregnant individuals in various settings and population groups. We informed our evaluation using established rigorous frameworks for identifying intervention characteristics (ie, the TIDieR framework) and for informing implementation design (ie, the CFIR). Eligibility criteria included usual care as the comparator group, which may increase generalizability to clinical settings.

This study also has several limitations, including a moderate to high risk of bias across most studies as previously reported^6^ and a lack in reporting of quality assurance measures, such as adherence or fidelity.^120^ There was also a lack of understanding of reach and capacity for implementation of lifestyle interventions in pregnancy, as previously reported.^120^ Included studies did not report against the TIDieR framework, so extraction of some components required subjective interpretation. In particular, there was limited information relating to gestational age at the completion of intervention or length of sessions (ie, minutes per hour), tailoring, adherence, or attrition rates, all of which limited our interpretative ability for the efficacy of these components in GWG reduction. As previously reported, significant publication bias was found against small studies reporting efficacy,^6^ which may have artificially increased the effect size. However our previous sensitivity analysis demonstrated negligible association with GWG efficacy, with studies deemed to be at low risk of bias.^6^ Subgroup analyses examining pooled effects by intervention type may have been underpowered.^126^

## Conclusions

This meta-analysis of randomized antenatal lifestyle interventions may advance the field by defining core and adaptable intervention components to underpin pragmatic implementation in routine pregnancy care as a critical next step to leverage the established efficacy and cost-effectiveness of interventions to optimize GWG and maternal and neonatal outcomes. We report broadly that lifestyle intervention delivery by an allied health professional appeared important with intervention content focused on diet and physical activity. Among dietary interventions, which were found to be associated with the greatest decrease in GWG in our previous study,^6^ those with an individual delivery format and moderate intensity were associated with the greatest change in GWG in this study. Physical activity and mixed behavioral interventions were beneficial but associated with less change in GWG; they therefore may benefit from earlier commencement and a longer duration for a more effective association with GWG reduction. These findings suggest that future pragmatic research should focus on testing and evaluating components to inform implementation in varied antenatal care settings, including those with limited resources, to optimize population benefit for pregnant individuals and the next generation.

## Notes

Supplement 1. **eFigure.** Diagram of Systematic Search **eTable 1.** Subgroup Analyses of 13 Diet Interventions in Pregnant Individuals on Gestational Weight Gain and Intervention Component **eTable 2.** Subgroup Analyses of 16 Diet With Physical Activity Interventions in Pregnant Individuals on Gestational Weight Gain and Intervention Components **eTable 3.** Subgroup Analyses of 42 Physical Activity Interventions in Pregnant Individuals on Gestational Weight Gain and Intervention Components **eTable 4.** Subgroup Analyses of 28 Mixed Interventions in Pregnant Individuals on Gestational Weight Gain and Intervention Components

Supplement 2.
Data Sharing Statement
