# Supplementary material for: Key Components of Antenatal Lifestyle Interventions to Optimize Gestational Weight Gain: Secondary Analysis of a Systematic Review
Source: JAMA Netw Open. 2023 Jun 16;6(6):e2318031. doi: 10.1001/jamanetworkopen.2023.18031 (PMC10276313; doi:10.1001/jamanetworkopen.2023.18031)
Supplement: Supplement 1. — eFigure. Diagram of Systematic Search eTable 1. Subgroup Analyses of 13 Diet Interventions in Pregnant Individuals on Gestational Weight Gain and Intervention Component eTable 2. Subgroup Analyses of 16 Diet With Physical Activity Interventions in Pregnant Individuals on Gestational Weight Gain and Intervention Components eTable 3. Subgroup Analyses of 42 Physical Activity Interventions in Pregnant Individuals on Gestational Weight Gain and Intervention Components eTable 4. Subgroup Analyses of 28 Mixed Interventions in Pregnant Individuals on Gestational Weight Gain and Intervention Components [file jamanetwopen-e2318031-s001.pdf]

Supplemental Online Content

Harrison CL, Bahri Khomami M, Enticott J, Thangaratinam S, Rogozińska E, Teede HJ. Key components of antenatal lifestyle interventions to optimize gestational weight gain. *JAMA Netw Open*. 2023;6(6):e2318031. doi:10.1001/jamanetworkopen.2023.18031

eFigure. Diagram of Systematic Search

eTable 1. Subgroup Analyses of 13 Diet Interventions in Pregnant Individuals on Gestational Weight Gain and Intervention Component

eTable 2. Subgroup Analyses of 16 Diet With Physical Activity Interventions in Pregnant Individuals on Gestational Weight Gain and Intervention Components

eTable 3. Subgroup Analyses of 42 Physical Activity Interventions in Pregnant Individuals on Gestational Weight Gain and Intervention Components

eTable 4. Subgroup Analyses of 28 Mixed Interventions in Pregnant Individuals on Gestational Weight Gain and Intervention Components

This supplemental material has been provided by the authors to give readers additional information about their work.

© 2023 Harrison CL et al. *JAMA Network Open.*

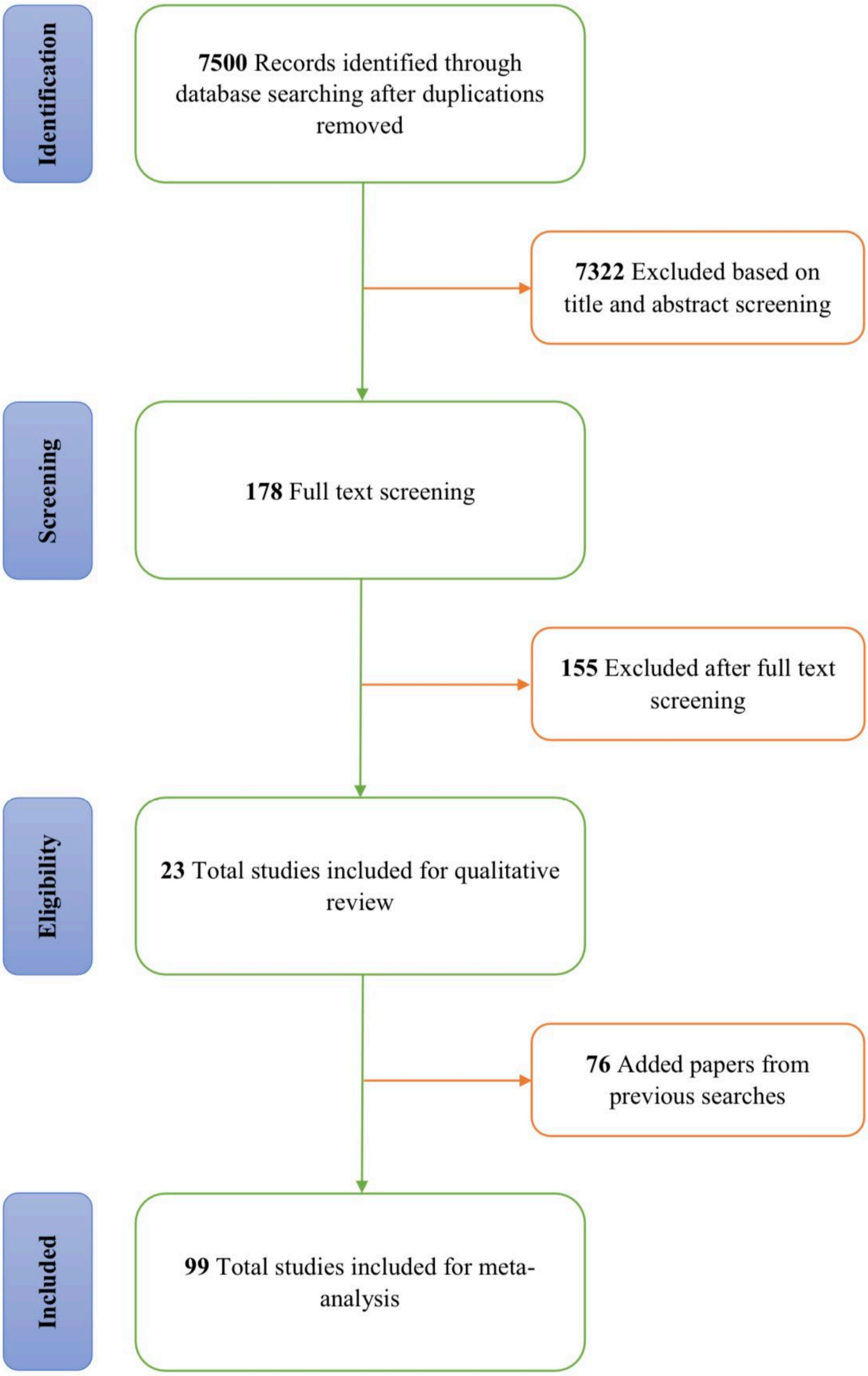

Figure 1. Preferred Reporting Items for Systematic reviews and Meta-Analyses (PRISMA) diagram of the systematic search (adapted from Teede et al 2022)

© 2023 Harrison CL et al. *JAMA Network Open.*

Supplementary table 1. Subgroup analyses of diet interventions (n=13) in pregnant women on gestational weight gain and TIDieR components.

		Studies (n)	Mean Difference, kg (95% CI)	I^2 (%)	p-Value for Subgroup Differences
**Theory**					0.03
	Theory based	3	-0.66 (-1.88 to 0.55)	80.5	
	Non-theory based/NR	10	-3.34 (-5.08 to -1.59)	95.3	
**Resources**					<0.001
	Self-monitoring tool	1	-9.07 (-10.90 to -7.24)	.	
	Other resource	6	-1.56 (-2.84 to -0.29)	89.3	
	Combination	0			
	None	6	-2.66 (-4.75 to -0.57)	94.2	
**Format**					0.002
	Individual	9	-3.91 (-5.82 to -2.01)	94.0	
	Group	4	-0.23 (-1.28 to 0.82)	85.6	
**Mode**					.
	Face to face	13	-2.63 (-3.86 to -1.40)	94.20	
	Remote	0			
	Face to face + Remote	0			
**Facilitator**					.
	Allied Health staff	13	-2.63 (-3.86 to -1.40)	94.20	
	Medical staff	0			
	Other	0			
	NA	0			
**Prior training**					0.94
	Yes	4	-2.70 (-4.53 to -0.87)	90.4	
	No/NR	9	-2.23 (-3.90 to -0.57)	95.4	
**Location**					.
	Hospital/Antenatal clinic	13	-2.63 (-3.86 to -1.40)	94.20	
	Exercise centre	0			
	Other	0			
**GA at commencement**					<0.001

© 2023 Harrison CL et al. *JAMA Network Open.*

	<20 weeks	10	-1.36 (-2.34 to -0.39)	88.9	
	>=20 weeks	2	-6.26 (-11.72 to -0.80)	95.2	
	NR	1	-6.80 (-8.63 to -4.97)	.	
**Duration**					<0.001
	High	3	-1.73 (-5.03 to 1.57)	84.1	
	Moderate	3	-0.89 (-1.61 to -0.16)	80.1	
	Low	2	-3.97 (-9.46 to 1.51)	98.2	
	NAC (insufficient data)	1	-6.78 (-8.41 to -5.15)	.	
**Number of sessions**					<0.001
	High	0			
	Moderate	4	-4.35 (-5.80 to -2.89)	45.9	
	Low	7	-0.50 (-1.23 to 0.24)	80.9	
	NR	2	-7.94 (-10.16 to -5.71)	66.2	
**Ongoing support**					0.14
	Yes	2	-1.31 (-2.39 to -0.22)	14.0	
	No/NR	11	-2.98 (-4.45 to -1.52)	95.1	
**Length of session**					0.83
	High	0			
	Moderate	2	-2.87 (-9.91 to 4.17)	93.3	
	Low	3	-1.87 (-7.54 to 3.81)	92.2	
	NR	8	-2.82 (-4.23 to -1.42)	94.5	
**Tailoring**					0.93
	Tailored	9	-2.66 (-4.16 to -1.16)	93.7	
	Not tailored/NR	4	-2.56 (-5.32 to 0.21)	96.1	
**Compliance**					0.96
	High	2	-2.87 (-9.91 to 4.17)	93.3	
	Low	0			
	NAC (insufficient data)	11	-2.72 (-4.07 to -1.38)	93.9	
**Attrition**					0.02
	High	1	-0.10 (-0.66 to 0.46)	.	
	Low	6	-2.55 (-4.04 to -1.07)	92.3	
	NAC (insufficient data)	6	-3.32 (-6.90 to 0.26)	95.9	

© 2023 Harrison CL et al. *JAMA Network Open.*

Supplementary table 2. Subgroup analyses of diet with physical activity interventions (n=16) in pregnant women on gestational weight gain and TIDieR components.

		Studies (n)	Mean Difference, kg (95% CI)	I^2 (%)	p-Value for Subgroup Differences
**Theory**					0.84
	Theory based	8	-1.40 (-2.32 to -0.48)	60.5	
	Non-theory based/NR	8	-1.27 (-2.13 to -0.42)	50.6	
**Resources**					0.01
	Self-monitoring tool	5	-0.68 (-2.51 to 1.150)	74.9	
	Other resource	2	-0.72 (-1.38 to -0.06)	0	
	Combination	6	-1.62 (-2.25 to -0.99)	0	
	None	3	-2.83 (-4.01 to -1.66)	0	
**Format**					0.77
	Individual	9	-1.41 (-2.36 to -0.46)	57.4	
	Group	7	-1.21 (-1.98 to -0.45)	48.8	
**Mode**					0.17
	Face to face	10	-1.37 (-2.16 to -0.58)	45.8	
	Remote	1	2.20 (-1.52 to 5.92)	.	
	Face to face+remote	5	-1.51 (-2.49 to -0.53)	67.3	
**Facilitator**					0.98
	Allied Health staff	13	-1.33 (-1.95 to -0.72)	42.9	
	Medical staff	2	-1.61 (-4.66 to 1.45)	91.0	
	Other	1	-1.32 (-3.99 to 1.35)	.	
	NA	0			
**Prior training**					0.10
	Yes	7	-0.87 (-1.56 to -0.18)	27.6	
	No/NR	9	-1.67 (-2.52 to -0.82)	55.9	
**Location**					0.53
	Hospital/Antenatal clinic	10	-1.54 (-2.42 to -0.66)	67.0	
	Exercise centre	4	-0.99 (-1.63 to -0.34)	0	
	Other	2	-0.16 (-4.01 to 3.70)	72.7	
**GA at commencement**					0.63

© 2023 Harrison CL et al. *JAMA Network Open.*

	<20 weeks	11	-1.24 (-2.04 to -0.43)	59.5	
	>=20 weeks	5	-1.55 (-2.54 to -0.56)	47.2	
	NR	0			
**Duration**					0.68
	High	4	-0.99 (-1.97 to -0.01)	0.0	
	Moderate	7	-1.60 (-2.63 to -0.57)	75.8	
	Low	5	-1.02 (-1.89 to -0.16)	4.9	
	NAC (insufficient data)	0			
**Number of sessions**					0.48
	High	3	-1.80 (-3.44 to -0.16)	73.3	
	Moderate	7	-0.94 (-1.61 to -0.27)	0.0	
	Low	6	-1.58 (-2.88 to -0.28)	72.4	
	NR	0			
**Ongoing support**					0.34
	Yes	6	-1.08 (2.00 to -0.17)	43.6	
	No/NR	10	-1.66 (-2.40 to -0.91)	57.6	
**Length of session**					0.19
	High	2	-0.26 (-1.22 to 0.70)	0	
	Moderate	5	-1.72 (-2.90 to -0.53)	54.5	
	Low	7	-0.91 (-1.43 to -0.39)	0.0	
	NR	2	-3.200 (-4.50 to -1.89)	0.0	
**Tailoring**					0.16
	Tailored	15	-1.26 (-1.90 to -0.63)	52.3	
	Not tailored/NR	1	-2.15 (-3.22 to -1.08)	.	
**Compliance**					0.65
	High	4	-1.43 (-2.11 to -0.76)	19.6	
	Low	4	-1.83 (-3.92 to 0.27)	63.7	
	NAC (insufficient data)	8	-0.10 (-1.83 to -0.16)	52.4	
**Attrition**					0.18
	High	2	-0.94 (-3.70 to 1.82)	77.4	
	Low	9	-1.02 (-1.73 to -0.30)	51.1	
	NAC (insufficient data)	5	-2.21 (-3.19 to -1.24)	11.3	

© 2023 Harrison CL et al. *JAMA Network Open.*

Supplementary table 3. Subgroup analyses of physical activity interventions (n=42) in pregnant women on gestational weight gain and TIDieR components.

		Studies (n)	Mean Difference, kg (95% CI)	I^2 (%)	p-Value for Subgroup Differences
**Theory**					<0.001
	Theory based	2	1.21 (0.20 to 2.23)	0.0	
	Non-theory based/NR	40	-1.16 (-1.43 to -0.89)	45.9	
**Resources**					0.35
	Self-monitoring tool	4	-1.30 (-2.74 to 0.14)	65.6	
	Other resource	5	-0.40 (-1.66 to 0.87)	61.6	
	Combination	3	-0.17 (-1.44 to 1.11)	0	
	None	30	-1.15 (1.45 to -0.85)	52.2	
**Format**					0.51
	Individual	7	-0.78 (-1.50 to -0.06)	0	
	Group	35	-1.06 (-1.38 to -0.74)	62.1	
**Mode**					.
	Face to face	42	-1.04 (-1.33 to -0.74)	56.2	
	Remote	0			
	Face to face+remote	0			
**Facilitator**					0.10
	Allied Health staff	30	-1.15 (-1.49 to -0.81)	59.6	
	Medical staff	6	-0.21 (-1.07 to 0.65)	45.6	
	Other	6	-1.51 (-2.07 to -0.94)	0.0	
	NA	0			
**Prior training**					0.14
	Yes	7	-0.65 (-1.14 to -0.16)	12.0	
	No/NR	35	-1.13 (-1.46 to -0.80)	58.2	
**Location**					0.92
	Hospital/Antenatal clinic	26	-1.10 (-1.42 to -0.78)	41.4	
	Exercise centre	11	-0.94 (-1.72 to -0.16)	78.4	
	Other	5	-1.15 (-1.87 to -0.44)	9.0	
**GA at commencement**					0.11

© 2023 Harrison CL et al. *JAMA Network Open.*

	<20 weeks	27	-1.26 (-1.50 to -1.01)	20.8	
	>=20 weeks	15	-0.65 (-1.36 to 0.06)	75.4	
	NR	0			
**Duration**					0.27
	High	20	-1.29 (-1.54 to -1.03)	32.2	
	Moderate	10	-0.38 (-1.58 to 0.81)	76.4	
	Low	10	-0.88 (-1.62 to -0.14)	55.0	
	NAC (insufficient data)	2	-0.30 (-2.34 to 1.74)	0	
**Number of sessions**					0.48
	High	35	-1.08 (-1.41 to -0.76)	61.8	
	Moderate	3	-0.27 (-1.22 to 0.67)	0.0	
	Low	3	-0.77 (-2.65 to 1.10)	0.0	
	NR	1	-1.12 (-2.12 to -0.12)	.	
**Ongoing support**					0.05
	Yes	4	0.12 (-1.05 to 1.28)	42.6	
	No/NR	38	-1.16 (-1.44 to -0.88)	49.5	
**Length of session**					0.52
	High	0		.	
	Moderate	34	-1.10 (-1.41 to -0.80)	53.4	
	Low	5	-0.65 (-2.22 to 0.91)	81.7	
	NR	3	-1.02 (-1.97 to -0.08)	0.0	
**Tailoring**					0.81
	Tailored	28	-1.00 (-1.35 to -0.65)	55.9	
	Not tailored/NR	14	-1.10 (-1.67 to -0.52)	58.1	
**Compliance**					0.25
	High	21	-1.26 (-1.63 to -0.89)	53.1	
	Low	10	-0.94 (-1.64 to -0.25)	57.0	
	NAC (insufficient data)	11	-0.59 (-1.28 to 0.11)	60.3	
**Attrition**					0.01
	High	7	-0.11 (-1.09 to 0.86)	49.2	
	Low	23	-1.38 (-1.68 to -1.08)	42.6	
	NAC (insufficient data)	12	-0.59 (-1.28 to 0.11)	66.3	

© 2023 Harrison CL et al. *JAMA Network Open.*

Supplementary table 4. Subgroup analyses of mixed interventions (n=28) in pregnant women on gestational weight gain and TIDieR components.

		Studies (n)	Mean Difference, kg (95% CI)	I^2 (%)	p-Value for Subgroup Differences
**Theory**					0.48
	Theory based	20	-0.66 (-1.02 to -0.30)	66.0	
	Non-theory based/NR	8	-0.96 (-1.73 to -0.20)	71.0	
**Resources**					0.44
	Self-monitoring tool	4	-0.55 (-1.13 to 0.03)	0.0	
	Other resource	9	-1.05 (-1.83 to -0.26)	60.2	
	Combination	14	-0.63 (-1.02 to -0.24)	72.3	
	None	1	-1.59 (-2.91 to -0.27)	.	
**Format**					0.10
	Individual	23	-0.60 (-0.92 to -0.28)	64.2	
	Group	5	-1.40 (-2.27 to -0.53)	58.5	
**Mode**					0.94
	Face to face	19	-0.83 (-1.23 to -0.44)	61.1	
	Remote	5	-0.52 (-1.73 to 0.69)	63.9	
	Face to face+remote	4	-0.74 (-1.42 to -0.06)	33.0	
**Facilitator**					0.06
	Allied Health staff	8	-0.71 (-1.27 to -0.15)	38.0	
	Medical staff	8	-1.10 (-1.88 to -0.32)	75.9	
	Other	7	-0.76 (-1.36 to -0.15)	51.6	
	NA	5	-0.25 (-0.98 to 0.48)	50.0	
**Prior training**					0.74
	Yes	14	-0.78 (-1.23 to -0.32)	67.0	
	No/NR	14	-0.74 (-1.25 to -0.23)	63.7	
**Location**					0.66
	Hospital/Antenatal clinic	19	-0.72 (-1.08 to -0.35)	59.2	
	Exercise centre	0			
	Other	9	-0.95 (-1.76 to -0.13)	67.3	
**GA at commencement**					0.33

© 2023 Harrison CL et al. *JAMA Network Open.*

	<20 weeks	19	-0.86 (-1.28 to -0.43)	61.1	
	>=20 weeks	9	-0.49 (-0.94 to -0.04)	55.5	
	NR	0			
**Duration**					0.66
	High	4	-0.84 (-1.39 to -0.28)	0.0	
	Moderate	15	-0.74 (-1.17 to -0.32)	74.6	
	Low	8	-0.76 (-1.19 to -0.33)	14.0	
	NAC (insufficient data)	1	0.76 (-1.73 to 3.26)	.	
**Number of sessions**					0.20
	High	2	-1.15 (-3.85 to 1.55)	66.7	
	Moderate	6	-0.81 (-1.40 to -0.21)	19.7	
	Low	17	-0.85 (-1.25 to -0.45)	63.5	
	NR	3	1.38 (-0.59 to 3.35)	20.6	
**Ongoing support**					0.79
	Yes	11	-0.74 (-1.20 to -0.27)	64.6	
	No/NR	17	-0.78 (-1.28 to -0.29)	67.7	
**Length of session**					0.10
	High	3	-1.90 (-2.90 to -0.89)	38.5	
	Moderate	6	-0.79 (-1.19 to -0.38)	0.0	
	Low	9	-0.74 (-1.33 to -0.15)	68.2	
	NR	10	-0.22 (-0.57 to 0.14)	32.3	
**Tailoring**					0.08
	Tailored	22	-0.56 (-0.90 to -0.22)	67.5	
	Not tailored/NR	6	-1.15 (-1.62 to -0.67)	8.6	
**Compliance**					<0.001
	High	7	-0.96 (-1.68 to -0.23)	66.0	
	Low	1	0.00 (-0.05 to 0.05)	.	
	NAC (insufficient data)	20	-0.77 (-1.17 to -0.38)	53.2	
**Attrition**					0.07
	High	5	-1.27 (-2.26 to -0.28)	52.1	
	Low	19	-0.47 (-0.77 to -0.16)	61.1	
	NAC (insufficient data)	4	-1.41 (-2.27 to -0.56)	39.6	

© 2023 Harrison CL et al. *JAMA Network Open.*
