# Supplementary material for: Key Components of Antenatal Lifestyle Interventions to Optimize Gestational Weight Gain: Secondary Analysis of a Systematic Review
Source: JAMA Netw Open. 2023 Jun 16;6(6):e2318031. doi: 10.1001/jamanetworkopen.2023.18031 (PMC10276313; doi:10.1001/jamanetworkopen.2023.18031)
Supplement: Supplement 2. — Data Sharing Statement [file jamanetwopen-e2318031-s002.pdf]

Data Sharing Statement

Harrison. Key Components of Antenatal Lifestyle Interventions to Optimize Gestational Weight Gain. *JAMA Netw Open*. Published online June 16, 2023. doi:10.1001/jamanetworkopen.2023.18031

Data
Data available: No
